# A commentary on the impacts of ‘Great Lockdown’ and its aftermath on scaling firms: What are the implications for entrepreneurial research?

**DOI:** 10.1177/0266242620961912

**Published:** 2020-11

**Authors:** Francis J Greene, Alessandro Rosiello

**Affiliations:** The University of Edinburgh, UK

**Keywords:** academic entrepreneurship, context, coronavirus pandemic, COVID-19, entrepreneurship policy, fast growth firms

## Abstract

This commentary argues that scaling fast growth firms drive economic development, even in recessionary periods. While the coronavirus induced the ‘Great Lockdown’ and its aftermath poses particular challenges, we argue that the crisis presents the entrepreneurial scholarly community with an opportunity to re-orientate our research. Rather than more narratives of business success in the face of adversity, the Great Lockdown presents us with a fresh opportunity to examine how scaling is affected by context, by luck and by the porous nature of business growth. In so doing, our hope is that it will encourage our community to adopt a more proactive agenda to support policy makers and entrepreneurs.

## Introduction

Scaling fast growth businesses are central to economic development; they create jobs, export, innovate and drive forward productivity gains even in recessionary periods (Greene, 2020; Mason, 2020; NESTA, 2011). However, the emergence of the coronavirus induced the ‘Great Lockdown’ and its aftermath is qualitatively different from earlier recessions, if only because of the way governments and institutions have directed entrepreneurial activity. Nonetheless, we predict that it will encourage entrepreneurial researchers to pursue a research agenda focused on the resilience of scaling firms. Concentrating only on how individual entrepreneurs cope, respond and succeed in spite of the pandemic represents, for us, a missed opportunity. Above all, the coronavirus crisis shows us how context – places, institutions, regulations – shape entrepreneurial activity. In this commentary, we argue for research agendas that more fully connect the micro with the meso and macro, for explanations that integrate luck and chance into understandings of business growth, and that provide a critical focus on why so few firms fail to scale.

Examining the porous nature of business growth has evident implications on how we engage with policy makers. Our standard approach has been to passively advise policy makers to provide particular forms of support for scaling firms. However, this approach looks increasingly questionable. To policy makers and business communities, we often appear rigorous and robust but lacking in relevance. We argue that the pandemic – alongside the fundamental challenges presented by climate change and declining levels of biodiversity – presents entrepreneurial researchers with the opportunity for a step change in how we can proactively engage and support policy makers and entrepreneurs.

## Why are scale-ups crucial to economic dynamism?

Entrepreneurship scholars have identified that scaling fast growth firms are crucial to economic development (Coad, 2009; Coad et al., 2014; Greene, 2020; Henrekson and Johansson, 2010). Typically, these are defined as firms with 10 or more employees that have seen businesses turnover and/or employment growth of more than 20% over a three-year period (Eurostat/OECD, 2007). Although they constitute only about 2%–6% of firms, they contribute about 50% of job creation and drive productivity growth (Du and Temouri, 2015; Haltiwanger et al., 2016). Such firms also associated with being more likely to export and innovate (Berthou and Vicard, 2015; Coad et al., 2016; Coad and Rao, 2008; Grazzi and Moschella, 2018). There are other identifiable features of scaling businesses; they tend to be younger and occur in all sectors rather than being concentrated in ‘high-tech’ ventures (Bravo-Biosca and Westlake, 2009; Daunfeldt et al., 2015; Henrekson and Johansson, 2010; Hölzl, 2009). However, the perennial problem with scale-ups is the difficulties in identifying them. Growth is spotty (Brown et al., 2014; Greene, 2020). Younger firms may only appear to grow faster because of the ‘up or out’ thesis – younger firms are more likely to grow rapidly than older firms but are also more likely to close than older firms (Haltiwanger, 2012; Pugsley et al., 2019). Few scale-ups show a consistent upwards pattern of growth. Instead, many experience scale-up growth episodes followed by downturns or even business closure. Daunfeldt and Halvarsson (2015), for example, describe Swedish fast growth firms as ‘one hit wonders’. These ephemeral qualities of scale-ups make them hard to categorise and risky to support as what looks like a scaling business today can turn into a zombie business tomorrow.

## How dynamic was small firm growth in the Great Recession of 2008?

Following from the last global recession of 2008, studies have explored the dynamism of small firm growth (Armand and Mendi, 2018; Bartz and Winkler, 2016; Fort et al., 2013; Peric and Vitezic, 2016). There are two basic hypotheses. First, smaller and younger firms exhibit faster growth because they are flexible and resilient and, consequently, find it easier to adapt their business models to recessionary conditions (Cowling et al., 2015). The alternative hypothesis is that smaller and younger firms struggle to grow due to their liabilities of smallness and newness, caused *inter alia* by difficulties in accessing finance, their limited customer range and reach, and their limited product or service portfolio (Davidsson and Gordon, 2016). In broad terms, studies show that the ‘Great Recession’ of 2008 had a negative impact on the growth potential of smaller and younger firms (Armand and Mendi, 2018; Bartz and Winkler, 2016; Fort et al., 2013; Peric and Vitezic, 2016). Scaling firms were not immune to the impacts of the 2008 recession. There were fewer start-ups and scale-ups (Anyadike-Danes and Hart, 2017; Klapper and Love, 2011; Sedláček and Sterk, 2017) and those that did start-up began smaller and remained smaller (Moreira, 2016). However, NESTA (2011) showed that although the numbers of fast growth firms dipped slightly over the period 2007–2010, they still provided around half of new jobs and had lower rates of insolvency than non-high growth firms. Equally, there is evidence that innovative firms were much more likely to be resilient after the 2008 recession (Amore, 2015; Cefis and Marsili, 2019).

## The scarring impacts of the Great Lockdown

Prior evidence though may be a poor guide for the vulnerability or resilience of scaling firms when faced with the coronavirus pandemic. Although we have been here before with other viruses (e.g. H1N1, H2N2, H3N2 and H1N1pdm09 viruses) – which hardly makes the coronavirus pandemic a ‘black swan’ event (Taleb, 2007) – the pandemic has been different from earlier recessions in the following four ways:

1. Government imposed lockdowns in many economies has led to predictions of a severe impact on the global economy. The Organisation of Economic Cooperation and Development (OECD; June 2020) predicted a 6% fall in global gross domestic product (GDP), while the World Bank (June 2020) predicted a contraction of 5.2%. By comparison, in 2009 the global economy shrank by just 0.1%.
  Some scenarios suggest that countries may bounce back relatively quickly from the pandemic (a ‘V’ shaped recovery). But others point to long term scarring patterns (‘L’ or ‘U’ shaped pattern) or – as with the Spanish flu epidemic of 1918–1920 – a ‘W’ shaped pattern whereby there are successive waves of the pandemic that interrupt economic activity (Jordà et al., 2020; Karlsson et al., 2014).
2. Supply *and* demand side small firm impacts. We have seen small firms face marked disruptions to their supply chains and their business networks. For Ireland, McCann and Myers (2020) identified that the pandemic has led to missed payments and uncertainties in both getting supplies and delivering them to customers. Unlike the 2008 recession, there have been direct impacts on both workers and entrepreneurs. Those working in small firms often have had to shift to remote working, juggle work with childcare, institute shielding practices to protect vulnerable family members while some sadly have had to deal with the consequences of becoming ill from the virus. The supply of entrepreneurial finance has also become more restricted. For example, an earlier commentary showed (Brown et al., 2020) equity finance is scarce, and where it is available it is often on less favourable terms (Mason, 2020).
  The Great Lockdown has also led to significant falls in business revenues. Greene and Rosiello (2020) found that two-third of UK firms had experienced a fall of turnover while German evidence showed that small firms had lost €75 billion in turnover in March 2020 alone (KfW, 2020). The pandemic affected particular sectors of the economy. Those such as tourism, construction, accommodation and food services saw dramatic falls in turnover. A lack of customer confidence and continued (localised) shutdowns increased the fragility of business revenues. These supply and demand pressures had immediate short-term impacts. To stem a liquidity crisis, many firms took on more debt and laid off staff or made them redundant; this did not prevent a sharp rise in business closures. In the United Kingdom, Prashar et al. (2020) found as a 70% increase in company dissolutions in March 2020, compared to March 2019; in the United States, Fairlie (2020) found that 22% of businesses were inactive due to the Great Lockdown.
3. Government control of the economy. To shore up the financial system after the 2008 financial crisis, it is estimated that the direct cost to the US economy was US$500 billion (Lucas, 2019). By comparison, the US initial government spend on coronavirus pandemic amelioration measures was US$1960 billion (Breugal, 2020). Governments have also imposed the forced closure of businesses and subsequently placed severe restrictions on how they do business. Not since the Second World War have governments assumed such a managerial role in capitalist economies. To ‘sugar the pill’, governments in wealthier economies supported businesses in four main ways. First, many took the unprecedented decision to fund the wage and income needs of both the employed and self-employed, extend sick leave entitlements and make it easier either to lay staff off temporarily or reduce their working hours. Governments also allowed businesses to defer tax and other payments to ease cashflow issues and – to support firm liquidity – introduced considerable and wide-ranging financial support in terms of loan guarantees, direct payments to firms and grants and subsidies. Finally, as countries began to come out of lockdown, governments introduced packages of support to retrain workers, to develop new markets and to help ease the transition to remote working.
4. New ways of working. The coronavirus pandemic led to the mass introduction of remote working and digitalisation. As with earlier recessions, there were some winners – businesses that were already operating successfully as an online business or were able to pivot to online (Arrighetti et al., 2016). Canadian survey evidence shows that some small firms accelerated their online sales but others struggled to adapt to e-commerce (CIBC, 2020). For some, this is impossible if they operate on a face-to-face basis. Even if they can shift to e-commerce, such transitions are difficult if the small firm lacks revenues and has limited access to external finance to fund these changes.

The pandemic, therefore, points to scaling being more difficult. Small firms are vulnerable to the deep and persistent scarring that the pandemic brings and many – including numerous viable scaling firms – have been swept away by the adverse economic impacts of the pandemic. Beyond temporary support, further government support is hedged in by their duty to repay the debts they have incurred to support businesses through the Great Lockdown.

## Out of a crisis comes opportunities

A less pessimistic appraisal of the pandemic is that it will create opportunities for individual firms to scale, and for the development of new sectors and ways of doing business. Entrepreneurial scholars may also see opportunities to pursue new research topics. One of these is entrepreneurial resilience (Herbane, 2010, 2019). Resilience from the Latin, resilire, literally means to bounce back (Sabatino, 2016). It is sometimes taken as the response to an external event, usually such as a natural disaster or, more broadly, how individual entrepreneurs remain resilient in the face of corruption or political instability (Dutta, 2017; Harries et al., 2018; Kwong et al., 2019; Williams and Shepherd, 2016). Alternatively, it is a process whereby scholars seek to understand the antecedents to the external shock or jolt; and how entrepreneurs subsequently cope, adapt and overcome these challenges (Bullough et al., 2014; Cheung and Kwong, 2017; Muñoz et al., 2019; Sine and David, 2003; Williams et al., 2017). These events and processes remain relatively lightly explored areas of scholarly investigation. Their appeal is that they follow the well-trodden approach of taking a person-centred agentic view of entrepreneurial endeavour that explains how individual entrepreneurs overcome the challenges they face. Subsequently, we can expect a stream of research about how new and existing firms faced the challenges of the pandemic and subsequently won through.

One fault line with this focus on resilience is that it represents the further reification of the entrepreneur. Because some of what passes as research in the study of entrepreneurship relies on purposive or convenience sampling, we can expect more histories of winners that identify the strategies used by entrepreneurs to pivot their business; for example, frugal management of resources, opportunity evaluation and enactment. This will generate yet more on how entrepreneurs learnt in the face of adversity, and what explains a ready association between stocks of resilience and entrepreneurial success (Battisti and Deakins, 2017; Battisti et al., 2019; Branicki et al., 2018; Ismail et al., 2011; Pal et al., 2014). In so doing, the role of luck is likely to be minimised. Growth is spotty or patchy both in good and bad times (Mason, 2020). Consequently, it is often more correct to talk of fast growth firms having episodes of growth rather than being intrinsically fast growth firms. This was particularly evident during the throes of the Great Lockdown. For example, demand for at home exercise solutions was steady before the lockdown; after the lockdown however, demand increased markedly. At the same time, gyms and personal fitness trainers saw their business evaporate overnight.

The pandemic has also shown us the quintessential importance of context. Welter (2011) has described contexts as a bundle of environmental assets and liabilities, with assets being conditions that support entrepreneurship while liabilities are stymie or impede entrepreneurial activities. Even before the pandemic, it was obvious that those economies with fertile entrepreneurial contexts produced better quality start-ups and supported scaling businesses (Baumol, 1990; Sanandaji and Leeson, 2013). In our discipline, there are those, particularly if they are rooted in economic geography, who point out that place contours business growth, with more dynamic regions producing higher scale-up rates (Brown et al., 2017; Mason and Brown, 2014). In the main, though, our discipline’s ‘go to’ approaches are rooted in Penrosean entrepreneurial strategy approaches such as the resource based view of the firm or those with a psychological turn that emphasise the relationships between, for example, individual entrepreneurial motivations or orientations and subsequent success (Bullough et al., 2014; Wales, 2016).

The Great Lockdown and its aftermath have shown the limited efficacy of these approaches to understanding entrepreneurial activities, behaviours and outcomes. By gazing only at the micro determinants of the firm, we miss the wider picture of how the individual entrepreneur connects with meso and macro environmental factors, and how entrepreneurial activity, processes and outcomes are shaped by institutions, place and temporality. We also neglect the role that luck plays in growth and how contextual opportunism (being in the right place, at the right time but having supportive institutions and fertile contexts) encourages entrepreneurship. Finally, in the rush to look at winners, we miss the vast majority who fail to grow their business.

The woes of the pandemic give us an opportunity to recast how we examine business growth. Instead of focusing on a tiny minority of episodic growers, a more holistic approach is to examine why growth is so hard to come by in particular contexts. Consequently, rather than looking at rare examples of success, we could examine why growth pipelines from early business start up onwards are so porous. This is a valuable endeavour because the reality is that the early promise of many firms ends up in either stalled growth, or in the disappointments of business closure.

## Policy implications

Shifting our focus also has implications for the manner in which we engage with policy makers and entrepreneurial communities such as entrepreneurs and support providers. Our standard ‘business model’ is to conduct robust and rigorous research and then write it up for publication, often after one or more rejections, in a journal. Our engagement generally consists of a section at the end of the article where we speculate on the policy and managerial implications of our findings. This section typically sets out what we would do if we had the opportunity to enact change; rarely does it indicate what the research has *done* to affect entrepreneurs or policy makers.

This passive observational approach makes some sense if the Great Lockdown and its aftermath pre-figure a further wave of Schumpeterian ‘creative destruction’. As such, we could see the pandemic as reinforcing and accelerating the shift towards digital entrepreneurship and the dominance of ‘big tech’, while simultaneously sweeping away swathes of small businesses that have traditionally focused on face-to-face personal service. A benign view of the pandemic therefore, is that it just another chapter of the evolution of capitalism as it moves through its cycle of prosperity, recession, depression and recovery. Consequently, the renting of the entrepreneurial fabric brought about by the pandemic in many rich and poor countries is likely to be a temporary jolt. Capitalism is resilient and will bounce back. If so, one neo-liberal response is to emphasise entrepreneurial agency and ignore the importance of context and of institutions in shaping entrepreneurial activities. After all, it is the agent rather than the structure that determines change. Another more neo-Keynesian response is to recommend a range of short-term and recycled support interventions to further tease out entrepreneurial successes. So, we could debate the necessary shape and size of continued financial support to help scaling firms deal with the ongoing impacts of the pandemic. Equally, we could wonder if business resilience programmes are patronising to firms that have already developed the resilience necessary to survive the pandemic.

These stances are a misreading of both Schumpeter and the pandemic. Schumpeter appears a proponent of laissez-faire capitalism because he saw depressions such as the great depression of the 1930s as having a necessary cleansing effect on capitalism (Schumpeter, 1941). This gives succour to the notion that the entrepreneur is to be set apart and to be applauded because their agency shapes our realities. However, Schumpeter (1939) also saw that some depressions were ‘pathological’ because ‘abnormal liquidation destroys many things which could and would have survived without it’ (p. 149). In such situations, Schumpeter (1934) argued for remedies to prevent the ‘abnormal course of events [that] are *really* meaningless and functionless’ (p. 236, emphasis in original). Schumpeter therefore, would surely have seen the Great Lockdown and its aftermath as ‘pathological’. Many of the responses to the pandemic have been radical; they have shown the centrality of institutions and contexts. Governments have not been passive referees, simply seeking to intervene only in the case of market failures; rather, they have sought to shape, direct and control economic activity.

So, what is our response as academics and researchers? We are part of the institutional fabric that generally supports and occasionally critiques entrepreneurship. The pandemic offers us the opportunity to shift our policy support from passive advice to proactive advocacy. Many of us work in business schools and we are often the most likely group of scholars within these schools to engage with businesses. And yet, a common complaint among both policy makers and entrepreneurs is that we might be robust and rigorous but we lack relevance. How then can we practically help scale-ups and policy makers? Traditionally, we have been good at pointing out the frailties of scale-up policy design and delivery, but how can we use our insights and the evidence base to provide relevant solutions?

We need not stop there. The pandemic is perhaps a spur for advocating radical policy directions. Arguably, the greatest challenge facing humans is the erosion of biodiversity and ongoing and altogether depressing impacts of man-made induced climate change on our environment. So what research agendas can we bring forward that will support new radical remedies? For instance, can we use our research to bring forward policies that will support scaling entrepreneurs to arrest or even reverse climate change? How can our evidence base better promote the viability, scalability and sustainability of circular economy business models? What can we do to support neglected communities if we want inclusive growth?
